# Impacts of Stress, Self-Efficacy, and Optimism on Suicide Ideation among Rehabilitation Patients with Acute Pesticide Poisoning

**DOI:** 10.1371/journal.pone.0118011

**Published:** 2015-02-13

**Authors:** Jun Feng, Shusheng Li, Huawen Chen

**Affiliations:** Department of Emergency Medicine, Tongji Hospital, Huazhong University of Science and Technology, Wuhan, China; University of Vienna, AUSTRIA

## Abstract

**Background:**

The high incidence of pesticide ingestion as a means to commit suicide is a critical public health problem. An important predictor of suicidal behavior is suicide ideation, which is related to stress. However, studies on how to defend against stress-induced suicidal thoughts are limited.

**Objective:**

This study explores the impact of stress on suicidal ideation by investigating the mediating effect of self-efficacy and dispositional optimism.

**Methods:**

Direct and indirect (via self-efficacy and dispositional optimism) effects of stress on suicidal ideation were investigated among 296 patients with acute pesticide poisoning from four general hospitals. For this purpose, structural equation modeling (SEM) and bootstrap method were used.

**Results:**

Results obtained using SEM and bootstrap method show that stress has a direct effect on suicide ideation. Furthermore, self-efficacy and dispositional optimism partially weakened the relationship between stress and suicidal ideation.

**Conclusion:**

The final model shows a significant relationship between stress and suicidal ideation through self-efficacy or dispositional optimism. The findings extended prior studies and provide enlightenment on how self-efficacy and optimism prevents stress-induced suicidal thoughts.

## Introduction

The use of pesticides for acts of suicide is recognized as a critical public health problem, especially in developing countries. Each year, at least 300 million people worldwide are estimated to suffer from severe acute pesticide poisoning. More than half of the victims reported the exposure was voluntary and was related to suicide [1]. Given its easy access, ingestion of pesticide has become the top means by which suicide is committed [2]. Certainly, suicide is such a complex event and multiple aspects that are involved in the process leading to the event should be considered. Suicide ideation and hopelessness have been identified as risk factors in suicidal behavior associated with pesticide ingestion [3]. Suicide ideation has been associated with organophosphous pesticide poisoning among banana workers in Costa Rica [4]. Scores for suicidal thoughts show a significant increase in risk for persons who had incidents of poisoning [5]. Farmers are often exposed to stressors such as workplace hazards caused by agriculture-related uncertainties and isolation, and thus, easy access to toxic substances may increase risk of injury or suicide [1]. The current study aims to explore the relationship between stress and suicide ideation and to identify protective factors for suicide ideation related to stress.

Research demonstrates that the ability to defend against the manifestation of suicidal thoughts is an important protective factor against suicidal behavior [6]. Adaptive cognitive characteristics, such as self-efficacy and dispositional optimism, are considered important factors in the protection from suicide ideation [6, 7]. Self-efficacy and optimism have been consistently related to health and functioning. Studies show that self-efficacy and dispositional optimism have significant effects on stress perception and on coping mechanisms in the fields of psychological and physical medicine [8–10]. Self-efficacy refers to the confidence and belief that an individual can act in a certain way for the purpose of meeting a goal or coping effectively in stressful situations [11]. Studies confirmed that self-efficacy has a significant effect on the behavioral style, effort, and persistence of an individual [12]. Studies also show that individuals with high self-efficacy show better health, achievement, and social integration [13]. People who have low self-efficacy usually feel powerlessness, in contrast to those with high self-efficacy who are more controlled and less threatened in stressful situations. This concept is also supported by another study that investigated the relationship between pain tolerance and stress. Results show that the experimental group had higher tolerance than the control group and placebo group after the individuals had increased cognitive control ability, which revealed that the sense of control had a safeguarding effect for individuals coping with negative stimulus [14]. In the preservation of psychological and physical health, self-efficacy could help in coping with stress and inducing positive effects in time. An inverse relationship between self-efficacy and acute stress reaction disorder has been found, which is also consistent with results of other studies [15]. Till et al demonstrated the associations between coping styles and individuals approaching toward films portraying the suicide of the protagonist [16]. They also found out that individuals tend to identify with the drama’s protagonist and tried to find out behavior patterns in the movie to against life events. In another words, according to Social-learning theory, imitations from films can also affect their favored coping strategies, enhance their self-efficacy and eventually defend against suicide behavior.

Theories of optimism are mainly focused on the future expectations of an individual [17, 18]. Optimism can be defined using two main scientific concepts, namely, dispositional and explanatory optimism [19]. Dispositional optimism was adopted in the present study. In general, optimists are peoples who believe that good things are more likely to happen to them than bad things. Optimists have positive expectations of the future. The concept of dispositional optimism was introduced by Scheier and Carver [20]; it is defined as generalized favorable expectations for one’s future outcomes. Studies show that optimists perform better than pessimists, especially in stressful situations [21, 22]. Given that optimists tend to hold positive expectations for their actions, they see these good outcomes as reachable, and they adopt behaviors that are helpful in achieving their goals. Studies found that optimists have lower depression scores than pessimists [21]. In addition, optimism is positively correlated with positive emotion and higher immunity [23]. Studies on stress coping styles also provide evidence that optimists tend to adopt problem-focused coping strategies, whereas pessimists are more likely to use self-approach, avoidance, and emotion-focused strategies [24]. Furthermore, optimism is a protective factor in physical and mental health, particularly against suicidal behavior caused by stress [25]. Research also shows that optimists are less likely to suffer from suicide ideation or to attempt suicide in cases of negative life events [7, 23].

Given that both self-efficacy and optimism assume the same psychological construct in evaluation of the future, a theoretical connection between these two factors has been proposed [26]. However, some important differences are found between these factors. High self-efficacy promotes positive expectations of the future, whereas low self-efficacy is associated with helplessness and pessimism [27]. As such, self-efficacy may be the cause or result of optimism; as supported by a research experiment that investigated the relationship between self-efficacy and a threatening stimulus. A threatening stimulus has a greater impact on participants with lower levels of self-efficacy, which eventually increases their levels of distress [28]. Another study compared different competing models among self-efficacy, optimism, social support, and happiness. Structural equation modeling (SEM) indicate that optimism partially mediated self-efficacy to subjective well-being (SWB) [18]. The model revealed significant paths from self-efficacy to SWB through optimism. The findings reveal a trend toward good outcomes for individuals with high scores on optimism and self-efficacy when exposed to risk factors for suicide. However, no study specifically investigated dispositional optimism and self-efficacy as mediating variables of stress events and suicide ideation. The present study seeks to enhance our understanding of the relationships among stress, self-efficacy, optimism, and suicide ideation among rehabilitation patients with acute pesticide poisoning. This study also aims to supplement previous research by addressing the concurrent effects of stress, self-efficacy, and optimism on suicide ideation. This research may help in the development of prevention and treatment programs that focus on promoting self-efficacy through holding positive expectations for their future as an effective response to stress.

## Methods

### 2.1 Ethics Statement

The institutional review board of the Huazhong University of Science and Technology approved all of the procedures in this study. The research background, purpose, and significance were relayed to all of the participants, who were asked to sign a written informed consent before research commenced.

### 2.2 Participants and Procedures

A total of 296 rehabilitation patients with acute pesticide poisoning from four large general hospitals in Wuhan, China were recruited for the study. Ages ranged from 23 years to 46 years, with a mean of 33.84 years (SD = 4.53); 68.92% of which were female (204) and 31.08% were male (92). Participants completed the study questionnaires in a classroom environment before they left the hospitals. Participants were informed that they were taking part in a psychological investigation with the purpose of knowing their psychological status, and there was no need to place their names on the measures. All participants have volunteered to take part in this study. Three hundred eight questionnaires were distributed, 12 of them were excluded from analysis because the participants did not completely answer the questionnaires. Participants received a gift worth ¥25 for compensation. Analysis was conducted using SPSS 16.0 and Amos 17.0.

### 2.3 Measures

Perceived Stress Scale (PSS) [29].

The PSS is a 14-item self-report questionnaire with the aim of assessing “the degree to which situations in people’s life are considered as stressful in the past month.” Psychological stress in this scale is defined as the degree to which an individual evaluates that the demands of a situation exceed one’s ability to cope. Examples of items include: “In the last month, how often have you been upset because of something that happened unexpectedly?” and “In the last month, how often have you felt that you were unable to control the important things in your life?” Likert 4-point scale, from 1 (not happened at all) to 4 (very often), was adopted in item-rating. PSS demonstrated adequate reliability with consistency Cronbach's α for .81, test-retest for .73 [28]. This scale has been frequently used in Chinese and is valid and reliable. In this study, the Cronbach alpha coefficient for the PSS was 0.879.

Generalized Self-efficacy Scale (GSES).

Zhang and his colleagues originally developed GSES in 1995 [30]. GSES is a self-report questionnaire that includes 10 items designed to measure self-efficacy in dealing with different problems such as novel experiences, stress, challenges, and pressure. Examples of items are: “I can always manage to solve difficult problems if I try hard enough.” and “If I am in trouble, I can usually think of a solution”. Likert 4-point scale, from 1 (not true at all) to 4 (very true), was adopted in item-rating. Studies have shown that this scale is valid and reliable for Chinese participants. In the sample from 23 nations, Cronbach's α ranged from 0.76 to 0.90 [29]. In our study, the Cronbach alpha coefficient of GSES was 0.863.

Life Orientation Test–Revised (LOT-R).

LOT-R was developed and revised by M. F. Sheier et al. [18,31], which is a 10-item self-report questionnaire. LOT-R is used to measure individual differences between generalized optimism and pessimism. Examples of items include: “If something can go wrong for me, it will.” and “It's easy for me to relax.” Likert 4-point scale, from 1 (disagree a lot) to 4 (agree a lot), was used in item-rating. The scale showed good reliability, with alpha coefficients of .84 for optimism and .86 for pessimism. Test-retest reliability over a two week period was .75 for optimism and .84 for pessimism [30]. In the present study, the Cronbach alpha coefficient was 0.857.

Beck Scales for Suicide Ideation (BSSI-C) [32].

The BSSI-C is a 19-item self-report measure of the intensity of specific attitudes, behaviors, and plans to commit suicide. The assessment includes criteria, such as, willingness to live, and active or passive suicidal desire. Items are rated from 1(never) to 3(very often). Examples of items include: “Passive suicidal desire.” and “Wish to die.” The total score of this scale can range from 0 to 38. Likert 5-point scale, from 1 (not happened at all) to 4 (very often), was adopted in item-rating. Test–retest coefficient for the BSSI-C was .54, average reliability coefficient for inpatients was .90, .87 for outpatients [31]. The BSSI-C was translated into Chinese and is reliable and valid. In the current study, the Cronbach alpha coefficient was 0.853.

### 2.4 Data Analysis

**Fig 1:**
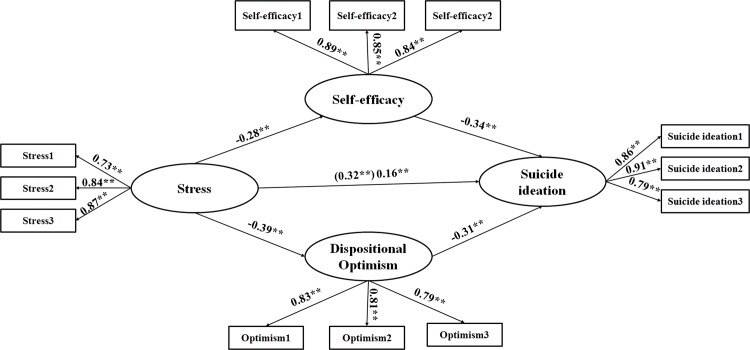
Final Structural Model (N = 296). Note: all factor loadings were standardized. Stress1–Stress3 = three parcels of stress; Self-efficacy 1–Self-efficacy 3 = three parcels of self-efficacy; Optimism1–Optimism3 = three parcels of dispositional optimism; Suicide ideation1–Suicide ideation3 = three parcels of suicide ideation; ** P<0.01

In this study, a model with two mediator variables was be tested (Fig. 1). To ensure the structural relationship of the latent structured model, a two-step modeling method suggested by Anderson and Gerbing [33] was adopted as following: Firstly, confirmatory factor analyzing measurement model was used to test accepted fit for the data; After which, the structural relationships among all the latent constructs were tested in the structural model. Anderson and Gerbing [32] concluded that the two-step approach has a number of comparative strengths included: First, it can test the significance of all pattern coefficients; second, this method allows an assessment of whether the structural models get acceptable fit or not; third, the asymptotically independent test can be made for the substantive or theoretical model of interest. Finally, the comparisons of several substantive models of interest can be provided. Maximum likelihood method in the Amos 17.0 was used to examine both CFA and structural models [34, 35].

To control the magnification of errors and improve the reliability and normality of the resulting measurements of personality [36, 37], parcels containing variables with no subscales were created [38]. Therefore, fewer indicators were used and data had better fit values. The mean of items in one parcel was used as an observed variable. Three item parcels were formed for all the variables using the factorial algorithm method proposed by Rogers and Schmitt [39].

The following indices recommended by Hu et al. [40, 41] were used to evaluate the goodness of fit of a model: (a) the standardized root mean square residual (SRMR, 0.08 or less), (b) the root mean square error of approximation (RMSEA,0.06 or less), and (c) the comparative fit index (CFI, 095 or greater).

## Results

### 3.1 Description and correlation analysis

Means, standard variance, and intercorrelation of all observed variables are presented in Table 1. Correlations between self-efficacy and dispositional optimism, as well as between suicide ideation and stress, were significant (P<0.01). Self-efficacy and dispositional optimism were negatively correlated with stress and suicide ideation (P<0.01).

**Table 1:** Intercorrelations between four latent variables.

	M	SD	1	2	3	4
1 Stress	2.52	0.64	1			
2 Self-efficacy	2.63	0.41	−0.31**	1		
3 Dispositional optimism	3.53	0.57	−0.24**	0.47**	1	
4 Suicide ideation	2.04	0.69	0.26**	−0.31**	−0.39**	1

Note: N = 296,

**, p<0.01.

### 3.2 Measurement Model

The purpose of CFA was to evaluate whether or not the model fit the data. The obtained indices show that the model fit the data well (see Table 2): χ^2^ (31, N = 296) = 166.58, P<0.001; RMSEA = 0.07, [0.05, 0.06]; SRMR = 0.07; and CFI = 0.96. In addition, all factor loadings for the nine measured variables on the latent variables were significant (P<0.001), showing that the four latent variables were well represented by their indicators.

**Table 2:** Fit indices of covariance structure analyses.

Model	χ^2^	df	CFI	SRMR	RMSEA	90% CI	Δχ^2^	Δdf
Measurement model	166.58	31	0.96	0.07	0.06	0.05–0.06	-	-
Partially mediated model (recommended model)	164.72	32	0.96	0.06	0.06	0.05–0.06	-	-
Direct Role	84.53	17	0.97	0.06	0.06	0.05–0.07	80.19	15
Fully model	165.74	33	0.98	0.06	0.06	0.05–0.06	1.02	1

### 3.3 Structural Model

Three steps were performed to find a suitable mediating structural model [18,42]. First, the direct effect of the predictor variable (stress) on the dependent variable (suicide ideation) was tested without the mediator. The direct standardized path coefficient was significant, β = 0.324, [0.216, 0.424], p <0.01. Second, a partially-mediated model (see Fig. 1), which included the mediator (self-efficacy and dispositional optimism) and direct paths, was tested in Amos 17.0. Results indicate that the model is a good fit for the data (see Table 2), χ^2^ (32, N = 396) = 164.72, p <0.01; RMSEA = 0.06, [0.05, 0.06]; SRMR = 0.06; and CFI = 0.96. Finally, a fully-mediated model that only tested the indirect effects was constructed; the indices indicated that the model fit the data: χ^2^ (33, N = 396) = 165.74, p <0.01; RMSEA = 0.06, [0.05, 0.06]; SRMR = 0.078; and CFI = 0.98. The Chi-square difference test shows no significant between the partially mediated model and the fully mediated model, χ^2^ (1, N = 396) = 1.02, (p = n.s.). Therefore, the partially mediated model was selected as the best representation of the data (Fig. 1). Taken together, results show the protective role of optimism and self-efficacy in the relationship between stress and suicide ideation. Although stress has a significant correlation with suicide ideation, optimism and self-efficacy mediate a positive relationship between them. The effects of stress on suicide ideation through self-efficacy and dispositional optimism were 25.52% and 28.09%, respectively.

### 3.4 Mediating Effect Testing

The mediating effect of self-efficacy or dispositional optimism on stress and suicide ideation was tested for significance by using the bootstrap estimation procedure in AMOS (a bootstrap sample of 1,200 was specified) [35]. According to Mackinnon et al. [43, 44], “the bootstrap method yields the most accurate confidence intervals for indirect effects”. Table 3 shows all the indirect and direct effects in 95% confidence intervals. As shown in Table 3, all of the indirect or direct effects in the partially mediated model show significance with 95% confidence interval and do not overlap with zero, which further provide evidence that the model is suitable.

**Table 3:** Direct and indirect effects and 95% confidence intervals for the final model.

Model pathways	Estimated effect	95% CI	
		Lower bonds	Up bonds
Direct effect			
Stress→Suicide ideation	0.161^a^	0.054	0.237
Stress→Self-efficacy	-0.284^a^	-0.419	-0.241
Stress→Dispositional Optimism	-0.387 ^a^	-0.518	-0.287
Self-efficacy→suicide ideation	-0.343 ^a^	-0.515	-0.241
Dispositional Optimism→suicide ideation	-0.309^a^	-0.469	-0.224
Indirect effect			
Stress→Self-efficacy→Suicide ideation	0.092^a^	0.014	0.141
Stress→Dispositional Optimism→Suicide ideation	0.116 ^a^	0.021	0.158

Note: ^a^ Empirical 95% confidence interval does not overlap with zero.

## Discussion

This study aimed to identify a model that includes self-efficacy and dispositional optimism as mediators in the relationship between stress and suicide ideation in patients with acute pesticide poisoning. SEM was used to determine the direct role of stress on suicide ideation, as well as the mediating roles of self-efficacy and dispositional optimism. Results show good level fit indices. In the partially mediated model, a positive relationship between stress and suicide ideation in patients with pesticide poisoning was found in the current study, which suggests that patients with higher stress levels were more likely to have higher levels of suicide ideation. Other studies also reported that daily exposure to stressors have a positive relationship with suicidal ideations [45, 46]. As mentioned previously, agriculture workers are more exposed to permanent stressful scenarios, such as low production and workplace hazards. Ecological studies have shown that agriculture workers with suicide ideation show statistically significant mortality compared with the general population; thus, psychologists or psychotherapists should pay more attention to the positive relationship between stress and suicide ideation [47]. The high incidence of pesticide poisoning, depression, injury, and suicide in farmers is an area of increasing concern [48].

The structural model also shows that the relationship between stress and suicide ideation weakened in patients with acute pesticide poisoning when self-efficacy and dispositional optimism are present. Results show that self-efficacy and optimism are partial mediators between stress and suicide ideation. That is, self-efficacy and optimism provide a buffer between stress and suicide ideation. In other words, self-efficacy and optimism have significant contributions in preventing suicide ideation caused by stress [7, 23]. In the present study, the mediating role of self-efficacy and optimism provides new insight on the relationship between stress and suicidal thoughts. Evidence from other studies [23, 49] shows that hopelessness in individuals increases levels of suicide ideation, in contrast to low levels of suicide ideation found in individuals with high levels of hope. In a follow-up study, King et al. [50] stated that the self-efficacy of a student lowered his suicidal tendency. As mentioned previously, both self-efficacy and dispositional optimism are cognitive constructs. Dispositional optimism is composed of positive expectations regarding future outcomes and self-efficacy is one’s belief in one’s ability to succeed in specific situations. Thus, people with high self-efficacy and optimism can combat stress better. As a result, self-efficacy and dispositional optimism have both direct and indirect roles in the correlation between stress and suicide ideation.

The results of this study suggest that optimism and self-efficacy provide protection from effects of stress in suicidal ideation. Moreover, studies found that high self-efficacy and optimism have a strongly negative effect on suicide ideation [51, 52]. The present study suggest that specific strengths (such as self-efficacy and optimism) may be important in understanding risk of suicide in an individual and provide potential means to ameliorate the risk. According to advocates of positive psychology, focusing on individual self-efficacy and optimism is important, which means that we can adopt interventions and counseling that promote self-efficacy and optimism to address suicide ideation. Research on suicide prevention education indicate that individuals can acquire skills and self-efficacy beliefs that enable them to adopt healthful behaviors when faced with suicidal factors [50]. People can benefit from positive expectations and modify their stereotypical patterns to have a positive perception of the future and themselves, which particularly coincide with Seligman’s concept of learned optimism [53], and thus, achieve behavior that discourages suicide.

This study has limitations. The data used in this study were collected from a certain patient group using cross-sectional methodology; thus, establishing a cause–effect relationship for the results obtained is difficult. Interpretation of the mediation effect of self-efficacy and optimism in the relationship between stress and suicide ideation should be treated with caution. Furthermore, self-report measures do not ensure accuracy of the responses of the participants. Thus, the generalization in this study may not be concurrent with that of other studies. Future longitudinal or experimental studies should be conducted to increase the generalizability of this study, so that the predictive power of stress for suicide ideation through self-efficacy or optimism would be more convincing.
